# Genetic Association and Expression Correlation between Colony-Stimulating Factor 1 Gene Encoding M-CSF and Adult-Onset Still's Disease

**DOI:** 10.1155/2020/8640719

**Published:** 2020-02-14

**Authors:** Yi-Ming Chen, Wei-Ting Hung, Wan-Chun Chang, Chia-Wei Hsieh, Wen-Hung Chung, Joung-Liang Lan, Ning-Rong Gung, Yun-Shien Lee, Der-Yuan Chen, Shuen-Iu Hung

**Affiliations:** ^1^Division of Allergy, Immunology and Rheumatology, Department of Medical Research, Taichung Veterans General Hospital, 407 Taichung, Taiwan; ^2^Faculty of Medicine, National Yang Ming University, 112 Taipei, Taiwan; ^3^Ph.D. Program in Translational Medicine, National Chung Hsing University, 402 Taichung, Taiwan; ^4^Institute of Clinical Medicine, National Yang Ming University, 112 Taipei, Taiwan; ^5^Department of Dermatology, Drug Hypersensitivity Clinical and Research Center, Linkou Chang Gung Hospital, 333 Taoyuan, Taiwan; ^6^School of Medicine, Chang Gung University, 333 Taoyuan, Taiwan; ^7^Cancer Vaccine and Immune Cell Therapy Core Laboratory, Department of Medical Research, Chang Gung Immunology Consortium, Chang Gung Memorial Hospital, Linkou, 333 Taoyuan, Taiwan; ^8^Whole-Genome Research Core Laboratory of Human Diseases, Chang Gung Memorial Hospital, 204 Keelung, Taiwan; ^9^Rheumatology and Immunology Center, China Medical University Hospital, 404 Taichung, Taiwan; ^10^School of Medicine, China Medical University, 404 Taichung, Taiwan; ^11^Rheumatic Diseases Research Laboratory, Rheumatology and Immunology Center, China Medical University Hospital, 404 Taichung, Taiwan; ^12^Department of Biotechnology, Ming Chuan University, 333 Taoyuan, Taiwan; ^13^Translational Medicine Laboratory, Rheumatology and Immunology Center, China Medical University Hospital, 404 Taichung, Taiwan; ^14^Institute of Pharmacology, National Yang-Ming University, 112 Taipei, Taiwan

## Abstract

Adult-onset Still's disease (AOSD) is a rare and inflammatory disorder characterized by spiking fever, rash, arthritis, and multisystemic involvement. HLA has been shown to be associated with AOSD; however, it could not explain the innate immunity and autoinflammatory characteristics of AOSD. To assess the genetic susceptibility of AOSD, we conducted a genome-wide association study (GWAS) on a cohort of 70 AOSD cases and 688 controls following a replication study of 36 cases and 200 controls and meta-analysis. The plasma concentrations of associated gene product were determined. The GWAS, replication, and combined sample analysis confirmed that SNP rs11102024 on 5′-upstream of *CSF1* encoding macrophage colony-stimulating factor (M-CSF) was associated with AOSD (*P* = 1.20 × 10^−8^, OR (95% CI): 3.28 (2.25~4.79)). Plasma levels of M-CSF increased in AOSD patients (*n* = 82, median: 9.31 pg/mL), particularly in the cases with activity score ≥ 6 (*n* = 42, 10.94 pg/mL), compared to the healthy donors (*n* = 68, 5.31 pg/mL) (*P* < 0.0001). Patients carrying rs11102024TT genotype had higher M-CSF levels (median: 20.28 pg/mL) than those with AA genotype (6.82 pg/mL) (*P* < 0.0001) or AT genotype (11.61 pg/mL) (*P* = 0.027). Patients with systemic pattern outcome were associated with elevated M-CSF and frequently observed in TT carriers. Our data suggest that genetic variants near *CSF1* are associated with AOSD and the rs11102024 T allele links to higher M-CSF levels and systemic outcome. These results provide a promising initiative for the early intervention and therapeutic target of AOSD. Further investigation is needed to have better understandings and the clinical implementation of genetic variants nearby *CSF1* in AOSD.

## 1. Introduction

Adult-onset Still's disease (AOSD) is an inflammatory disorder characterized by spiking fever, macular rash, leukocytosis, arthritis, variable multisystemic involvement, and increase of acute phase reactants [1, 2]. It is a rare disease with crude prevalence of only 1-2 cases in 100,000-1 million annually [3, 4]. The clinical features and disease progression of AOSD vary considerably. In severe cases, AOSD may lend to permanent joint destruction, organomegaly, lymphadenopathy, serositis, and aseptic meningitis [5–7]. Due to its characteristics and predominate dysregulation of innate immunity, AOSD has been considered an autoinflammatory disease [8, 9]. Standard treatments for AOSD include corticosteroids as the first-line, the use of nonsteroidal anti-inflammatory drugs (NSAIDs), and disease-modifying antirheumatic drugs (DMARDS) to manage the clinical symptoms [6, 10, 11]. Nevertheless, AOSD still lacks effective therapeutics, as its etiology and pathophysiology remain unclear [12].

The proposed pathomechanisms of AOSD involve the dysregulation of the immune system [13, 14], interaction between host and environment factors [15–17], and genetic susceptibility [18–21]. Important laboratory characteristics of AOSD are leukocytosis with predominance of neutrophils and the increase of proinflammatory cytokines, including IL-1*β*, IL-6, IL-18, TNF-*α*, and IFN*γ*, but with negative testing for rheumatoid factor (RF) and autoantibodies [14, 22, 23]. In particular, macrophage activation is a clinic feature of AOSD [8, 24]. High levels of macrophage colony-stimulating factor (M-CSF), a critical growth factor for macrophage differentiation and activation, have been observed in the plasma of AOSD patients [22, 25, 26]. The heterogeneous phenotypes and biosignature of AOSD imply the potential involvement of complex genetic predisposition.

Most of the previous genetic studies on AOSD revealed association with variants on human leukocyte antigen (*HLA*) class I and II regions, such as *HLA-BW35*, *HLA-DRB1*∗*15* and *HLA-DRB1*∗*04* [19, 20]. However, the association was inconsistent and controversial among the various studies of different populations [27, 28]. In addition, the results of genetic studies did not link to the pathogenesis, and none of them have been associated with AOSD disease outcomes [14, 28]. Here, we enrolled 106 patients and 888 population controls and applied GWAS discovery and subsequent replication analysis to investigate the genetic susceptibility of AOSD. We explored the correlation between the identified genetic risk factor(s) and disease severity or outcomes and examined the functional implication of the associated genetic variants in AOSD.

## 2. Materials and Methods

### 2.1. Patient and Public Involvement

This study was carried out following the rules of the Declaration of Helsinki of 1975, which was revised in 2013. This study was approved by the ethics committee of the Institutional Review Board (IRB) of Taichung Veterans General Hospital (CF13321), and the written consent was obtained from each participant. We enrolled a total of 106 AOSD patients fulfilling the Yamaguchi criteria [29] between January 2010 and December 2015. Patients with infections, malignancies, or other rheumatic diseases were excluded. The disease activity of AOSD was assessed with a modified Pouchot score described by Rau et al. [23]. According to the proposed classification of disease courses of AOSD [9], the AOSD patients with follow-up at least one year were classified into two patterns of disease outcomes: (i) the “systemic pattern” that includes the monocyclic and the polycyclic form and (ii) the “chronic articular pattern” (persistent arthritis involving at least one joint destruction and lasting longer than 6 months) [30]. All of the AOSD patients were unrelated Han Chinese. Seventy of them were randomly selected as the case group in the GWAS discovery cohort.

We recruited 924 ethnically and geographically matched healthy subjects as the population controls from a biobank under a nationwide population study, which comprises 9,980 Han Chinese descendants [31]. There was no self-report of rheumatic diseases among the recruited controls from Taiwan, where 98% of the population is made up of Han Chinese. Of the 924 population controls, we randomly obtained 724 controls for the GWAS discovery cohort and the rest of 200 individuals were for the replication cohort, which was an independent analysis conducted by 36 AOSD cases versus 200 population controls to validate the statistically significant SNPs derived from the initial GWAS results.

### 2.2. Genotyping and Quality Controls in the Genome-Wide Scan

Genomic DNA was extracted from the peripheral blood of the enrolled subjects using Flexi Gene DNA kits (Qiagen, Hilden, Germany). We performed GWAS on the samples obtained from 70 AOSD cases and 724 population controls using the Affymetrix SNPs Array 6.0 platform (Santa Clara, CA, USA), which is composed of 909,622 SNPs [31]. Briefly, 200 ng of genomic DNA of each sample was PCR amplified, fragmented, precipitated, and resuspended in the appropriate hybridization buffer. After hybridization, the BeadChip oligonucleotides were extended by a single-labeled base, which were detected by fluorescence imaging with an Affymetrix Bead Array Reader. Normalized bead intensity data obtained for each sample were loaded into the Affymetrix SNP Array 6.0 software, which converts the fluorescence intensities into SNP genotypes. The genotype calls were generated using the Birdseed method (Birdseed v2) with Affymetrix Power Tools (version, apt-1.10.2).

We analyzed the GWAS data by the software Plink (v1.90b5) using logistic regression modeling with covariates: sex, and ancestry-specific principal components (i.e., PC1, PC2, PC3, PC4, PC5, PC6, PC7, PC8, PC9, and PC10). The genomic coordinates are based on NCBI Human Genome Build 37 (GRCh37). Of the 909,622 genotyped SNPs, 868,494 SNPs located on the autosomal chromosome were included for the quality control (QC) process. Our quality control for SNPs was a call rate higher than 0.95, and 59,494 variants were removed due to missing genotype data. We excluded 163,017 variants due to minor allele threshold(s) (minor allele frequency (MAF) < 0.01). The quantile-quantile plot was generated using the R/Bioconductor package: GWASTools [32]. The genomic inflation factor was calculated by Plink (v1.90b5). Linkage disequilibrium (LD) analysis of the SNP rs11102024 was performed by using the LDproxy module of the online software package LDLink (https://analysistools.nci.nih.gov/LDlink).

### 2.3. Replication Analysis and Targeted Gene Sequencing

For the replication study, we applied TaqMan assays (Thermo Fisher Scientific, CA, USA) or direct sequencing on the associated SNPs revealed by the initial GWAS using an independent sample set of 36 AOSD cases and 200 population controls. The oligonucleotide primers for polymerase chain reaction (PCR) amplification of the SNP rs11102024 near *CSF1* gene are forward primer: 5′-TCCTATTGCATTGGGCATATT-3′, and reverse primer: 5′-TCCATTTACGCC TCAACTCA-3′, and those for SNP rs9636107 near *TCF4* gene are forward primer: 5′-GCTGGTACCAAGGAAAG CTG-3′, and reverse primer: 5′-CCTGCTGGTGTTTTGTTTTG-3′. The PCR reaction was performed in three steps: 3 min at 95°C; then 40 cycles of 20 seconds at 95°C, 30 seconds at 58°C, and 30 seconds at 72°C, followed by 7 minutes at 72°C.

### 2.4. Determination of Plasma Levels of Macrophage Colony-Stimulating Factor (M-CSF)

The plasma samples of 82 patients with AOSD were collected at the active status of the disease, and the patients received corticosteroids and/or the nonsteroidal anti-inflammatory drugs (NSAIDs). Besides, the disease-modifying antirheumatic drugs (DMARDs) had also been prescribed for the patients, which included methotrexate (patients number (*n*) = 74), hydroxychloroquine (*n* = 66), cyclosporine (*n* = 30), sulfasalazine (*n* = 18), and azathioprine (*n* = 10). Levels of plasma M-CSF were determined on the samples from these 82 active AOSD patients and 68 population controls using enzyme-linked immunosorbent assay (ELISA) according to the manufacturer's instructions (Ray-Biotech Inc., GA, USA).

### 2.5. Statistical Analysis

For the GWAS, we conducted the statistical analysis for the associations by comparing the allele frequencies between AOSD cases and population controls. The SNP association was examined by Fisher's exact tests and rank-ordered according to the lowest *P* value. All the *P* values were two-tailed. The corrected Pc values were adjusted using Bonferroni's correction for multiple comparisons (645,983 for GWAS SNPs), and Pc value for genome-wide significance should be <7.8 × 10^−8^ (0.05/645,983). The odds ratios (OR) were calculated using Haldane's modification [33]. Levels of plasma M-CSF in the different groups were compared by the nonparametric Mann-Whitney *U* test. The differences in the frequencies of significant alleles among AOSD patients with different courses of disease outcomes were examined using Fisher's exact test. The correlation coefficient was obtained by nonparametric Spearman's rank correlation test. A probability of less than 0.05 was considered to be significant.

## 3. Results

### 3.1. Clinical Characteristics of AOSD Patients

The demographic data, clinical characteristics, and laboratory findings of 106 patients with AOSD are shown in Table 1. The mean age of the patients is 42.9 years (standard deviation (SD): 15.0), and the proportion of females is 67.9%. The presence of spiking fevers (≥39°C) is observed in 102 (96.2%) cases, evanescent rash in 94 (88.7%) cases, arthralgia or arthritis in 78 (73.6%) cases, sore throat in 74 (69.8%) cases, liver dysfunction in 42 (39.6%) cases, lymphadenopathy in 39 (36.8%) cases, and hepatosplenomegaly shown by sonography in 14 (13.2%) cases, respectively. The mean of white blood cell count in the 102 patients is observed as 16,205 cells/microliter (SD: 11673), platelet count as 351.5 × 10 [3]/cumm (SD: 151.0), ESR (erythrocyte sedimentation rate) value as 78.4 mm/1st hour (SD: 36.6), CRP (C-reactive protein) as 10.2 mg/dL (SD: 7.8), ferritin level as 8989 *μ*g/L (SD: 15328), and clinical activity score as 5.48 (SD: 1.11).

### 3.2. Genome-Wide Scan of AOSD

We first conducted a GWAS using samples of 70 AOSD patients and 724 controls. After the QC process, 36 controls were removed, and 645,983 variants and 758 samples (including 70 cases and 688 population controls) passed the filters. The mean call rate is 98.3%. The SNPs on sex chromosomes were excluded from the GWAS analysis. The principal component analysis (PCA) map showed that there is no difference in the distribution of ancestry among AOSD cases and population controls (Supplemental [Supplementary-material supplementary-material-1]). A quantile-quantile plot of the test statistics was used for quality control and revealed that the population matching was successful (Supplemental [Supplementary-material supplementary-material-1]). The genomic inflation factor (*λ*GC) [34] is 1.05, suggesting that the population structure of our GWAS is generally acceptable.

**Figure 1:**
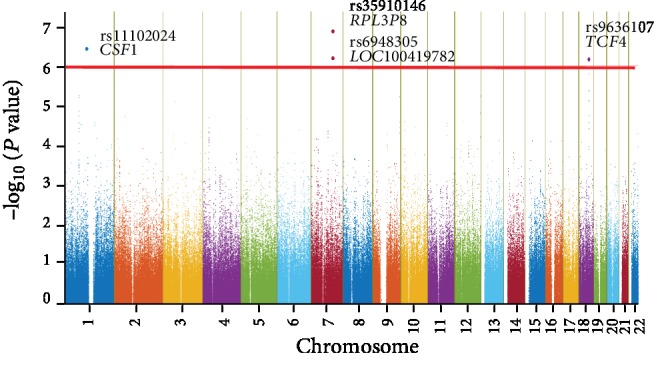
Genome-wide association scan for AOSD. Manhattan plot shows association between 645,983 SNPs and AOSD. Each dot represents a –log_10_ (*P* value) derived with a logistic regression model adjusted by sex and principal components (PCs) in 70 AOSD cases and 688 population controls. The red horizontal line marks the level of significance suggestive of association (*P* < 1 × 10^−6^). The top 4 AOSD-associated variants are labeled with the name of the nearest gene(s). *CSF1*: colony-stimulating factor 1; *RPL3P8*: ribosomal protein L3 pseudogene 8; *LOC100419782*: pseudogene; *TCF4*: transcription factor 4.

When comparing the allele frequencies of the 645,983 SNPs of 70 AOSD cases and 688 population controls, no SNP reached the threshold of genome-wide significance (Pc < 7.8 × 10^−8^). Given AOSD is a rare disease and small sample size used in this study, we set the cut off *P* value as 1 × 10^−6^ and the top 4 significant SNPs with highly suggestive association are shown in Figure 1 and Table 2. Of these 4 SNPs, two SNPs (rs35910146 and rs6948305) are on the upstream of pseudogenes, and rs9636107 does not match Hardy-Weinberg equilibrium (*P* = 0.03) in the dataset of 688 population controls (Table 2). The SNP rs11102024 on 5′-upstream of *CSF1* (colony-stimulating factor 1) gene encoding for M-CSF (macrophage colony-stimulating factor) on chromosome 1p13 showed significant association with AOSD (*P* = 3.70 × 10^−7^, OR (95% CI): 3.27 (2.07~5.17)) (Table 2). By comparison, the association strength between SNPs on HLA regions and AOSD was weaker than that of variants near *CSF1* gene (Supplemental [Supplementary-material supplementary-material-1]).

### 3.3. Replication, Meta-Analysis, and Linkage Disequilibrium of Variants near *CSF1* Gene

**Figure 2:**
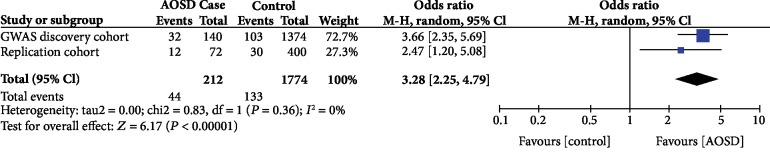
Meta-analysis of the genetic association between rs11102024 and adult-onset Still's disease (AOSD). M-H: Mantel-Haenszel test.

We used the samples of an independent cohort (36 AOSD cases and 200 controls) to replicate the association. Among the 4 SNPs discovered by GWAS, only SNP rs11102024 near *CSF1* displayed significant association with AOSD (*P* = 0.022, OR (95%CI) = 2.47 (1.20~5.08)) (Table 3). The meta-analysis of the two datasets from the initial GWAS and replication revealed that the *P* value of the heterogeneity test between studies (*P*_het_) was 0.36, suggesting that there was no difference between both studies (*P* value of the heterogeneity test > 0.05) (Figure 2, Table 3). Subsequently, we performed combined sample analysis using the genotyping data of 106 AOSD cases and 888 population controls, and the SNP rs11102024 showed strong association with AOSD (*P* = 1.20 × 10^−8^; OR (95% CI): 3.28 (2.25~4.79)) (Table 3, Figure 2). We used the genetic dataset of East Asian population of the 1,000 genome project for linkage disequilibrium (LD) analysis and identified 11 SNPs having strong LD with rs11102024 (*r*^2^ > 0.7) (Supplemental [Supplementary-material supplementary-material-1], Supplemental [Supplementary-material supplementary-material-1]). We also sequenced the *CSF1* gene; however, there were no missense SNPs with significant association with AOSD (data not shown).

### 3.4. Increased Plasma M-CSF Levels in AOSD Patients and Correlation with the Activity Score of the Disease

**Figure 3:**
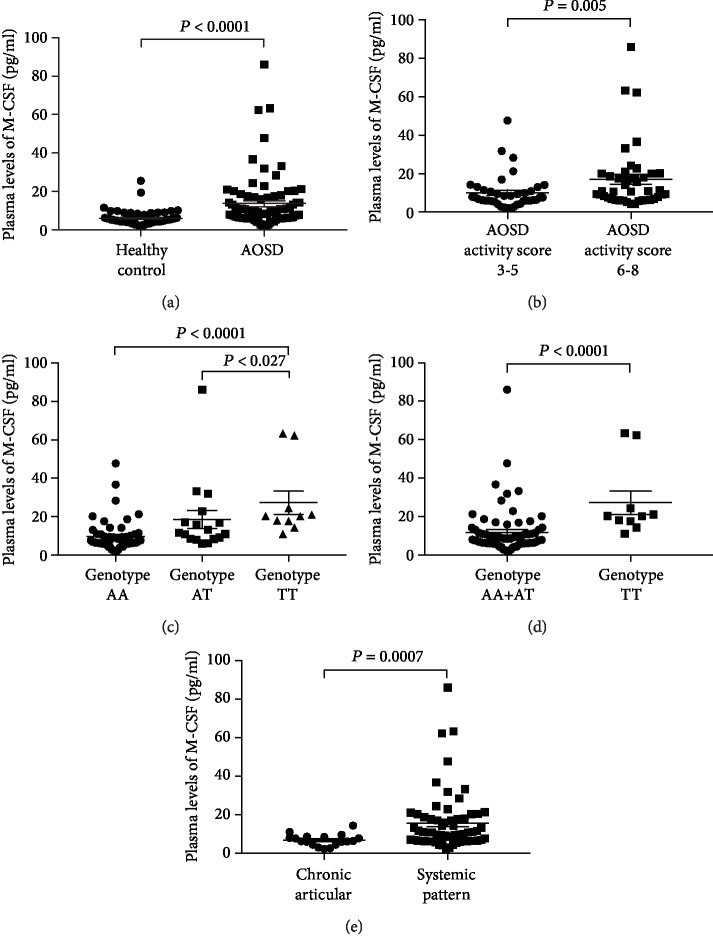
Increase of plasma M-CSF (macrophage colony-stimulating factor) levels in AOSD patients and correlation with the activity score, disease outcomes, and genotypes of SNP rs11102024 nearby *CSF1* gene. (a, b) The plasma levels of M-CSF protein were determined in the samples of healthy controls (*n* = 68) and AOSD patients (*n* = 82). (a) A significant increase of plasma M-CSF levels was detected in the AOSD patients compared to those of healthy controls. (b) The plasma levels of M-CSF are significantly increased in AOSD patients with activity scores 6-8, compared to those of AOSD patients with activity scores 3-5. (c, d) The AOSD patients (*n* = 82) were divided into three groups according to the genotype of rs11102024. (c) AOSD patients carrying the SNP rs11102024 TT genotype showed significantly higher levels of M-CSF (*n* = 10, median: 20.28 pg/mL), compared to the carriers of AA genotype (*n* = 55; median: 6.82 pg/mL) (*P* < 0.0001) and AT genotype (*n* = 17; median: 11.61 pg/mL) (*P* = 0.027). (d) A significant increase of plasma M-CSF levels was detected in AOSD patients with rs11102024 TT genotype than those of AA/AT genotypes. (e) Correlation between the plasma levels of M-CSF and disease outcome of 82 AOSD patients (systemic pattern (*n* = 64) and chronic articular pattern (*n* = 18)). AOSD patients with the systemic pattern showed a significant increase of M-CSF (median: 10.79 pg/mL) compared to those with chronic articular pattern (median: 6.23 pg/mL). These results are expressed as the mean ± SEM with each dot representing the data of an individual. Statistical analysis was performed using a two-tailed, nonparametric Mann-Whitney *U* test.

We hypothesized that genetic variant near *CSF1* might have an impact on the disease progress/outcome of AOSD. We determined the plasma levels of M-CSF in 82 patients who had available plasma samples. Significantly higher levels of plasma M-CSF were detected in AOSD patients (median 9.31 pg/mL, inter-quartile range (IQR) 6.21-16.91 pg/mL) compared with the healthy controls (*n* = 68) (median 5.31 pg/mL, IQR 4.12-6.85 pg/mL) (nonparametric Mann-Whitney *U* test, *P* < 0.0001) (Figure 3(a)). Moreover, significantly higher M-CSF levels were detected in the AOSD patients with activity score ≥ 6 (*n* = 42; median 10.94 pg/mL; IQR 6.73-20.23 pg/mL), compared to those with activity score between 3 and 5 (*n* = 40; 7.68 pg/mL, IQR 5.88-11.38 pg/mL) (Figure 3(b)).

### 3.5. Correlation of Plasma M-CSF Levels, the Genotypes of *CSF1*, and Disease Outcome of AOSD

The AOSD patients (*n* = 82) were divided into three groups according to the genotype of rs11102024. The highest M-CSF levels were observed in AOSD patients carrying the TT genotype (*n* = 10; median: 20.28 pg/mL; IQR 16.78-33.86 pg/mL) compared with the carriers of AA genotype (*n* = 55; median: 6.82 pg/mL; IQR 5.88-10.79 pg/mL) (nonparametric Mann-Whitney *U* test, *P* < 0.0001) and AT genotype (*n* = 17; median: 11.61 pg/mL, IQR 8.47-19.98 pg/mL) (*P* = 0.027), respectively (Figure 3(c)). Furthermore, AOSD patients carrying the rs11102024 TT genotype displayed significantly higher M-CSF expressions (*n* = 10; median: 20.28 pg/mL; IQR 16.78-33.86 pg/mL) than those with AA/AT genotypes (*n* = 72; 7.95 pg/mL, IQR 6.03-12.73 pg/mL) (*P* < 0.0001) (Figure 3(d)).

Regarding the disease outcome, 64 of 82 AOSD patients had the systemic pattern, and 18 cases had the chronic articular pattern. Significantly higher M-CSF levels were observed in AOSD patients with the systemic pattern (median 10.79 pg/mL, IQR 6.40-18.53 pg/mL) compared to those with the chronic articular pattern (6.23 pg/mL, IQR 4.37-8.41 pg/mL) (*P* = 0.0007) (Figure 3(e)). In addition, the AOSD patients with the systemic pattern accounted for 80% of the rs11102024 TT genotype carriers, comparing to only 20% with the chronic articular pattern.

## 4. Discussion

AOSD possesses similar clinical presentations with rheumatoid arthritis (RA); however, AOSD does not have RF or significant memorial lymphocyte involvement, and the genetic susceptibility revealed by this study showing no similarity with that of rheumatoid arthritis [35]. Consistent with the previous study, we did not find association between AOSD and *MEFV* gene mutations [36–38] or other genetic mutations of monogenic autoinflammatory disorders [39, 40]. The systemic juvenile idiopathic arthritis (juvenile-onset Still's disease) has multiple-gene predisposition [8, 41], including *HLA-DRB1* [21, 42–44]. In addition, both HLA class I and II regions have been reported to be risk loci for AOSD [29]. However, these findings are controversial to the clinical characteristics of juvenile-onset or adult-onset Still's disease, as HLA would implicate the predominant adaptive immune response. In this study, we find that SNPs on HLA class I and class II had only weak association with AOSD, but 5′-upstream variants of *CSF1* reached genome-wide significance. The different observations may relate to the enrolling criteria for patients with diverse clinical presentations of Still's disease [18, 44]. Further studies are needed to verify these genetic associations with AOSD in different populations.

*CSF1* gene encoding M-CSF has been implicated in the differentiation and activation of monocyte/macrophage lineage [25, 26]. The increased M-CSF plasma levels have been considered a biomarker for AOSD [22, 25, 26]. In our study, M-CSF expression correlating with AOSD activity and severity is consistent with the results of previous studies [22, 25, 26]. Particularly, our GWAS first demonstrates that SNP rs11102024 on 5′-upstream of *CSF1* reached genome-wide significance, and the subsequently replication study confirmed the strong genetic association with AOSD. We further identified 11 SNPs having strong LD with rs11102024 (*r*^2^ > 0.7), and most of them show regulatory potential [45]. As the associated SNP rs11102024 is only 22 kb upstream of *CSF1* and the nearby LD variants show regulatory potential, we propose that these SNPs may upregulate the expression levels of M-CSF, resulting in the disease of AOSD. In support of the functional roles of associated genetic variants, we indeed found significantly higher levels of plasma M-CSF in our AOSD patients with rs11102024 TT genotype. Taken together, our data suggest that the variants near *CSF1* may play important pathogenesis roles to regulate the gene expression and participate in the disease course of AOSD. Further studies are needed to investigate the potential mechanism of action of the genetic variants in the context of AOSD.

The clinical course of AOSD can be divided into two main patterns with different prognoses: systemic pattern (monophasic or polycyclic) and chronic articular pattern. The systemic pattern is characterized by predominantly systemic features including fever, rash, serositis, and organomegaly with or without articular symptomatology. By comparison, the chronic articular pattern is characterized by the severe articular manifestations mimicking rheumatoid arthritis. In this study, we observed higher plasma levels of M-CSF in AOSD patients whose activity score ≥ 6, cases with systemic pattern, or carriers with rs11102024 TT genotype. These data suggest that rs11102024 T allele, which is highly associated with M-CSF plasma levels, could be used to predict the severity and system symptoms in the early phase of AOSD and act as a promising genetic marker for early intervention to improve AOSD outcome.

As AOSD is a polygenetic autoinflammatory disorder, increasing biologic agents are investigated to target its proinflammatory cytokines, such as IL-1 family (particularly IL-1*β* and IL-18), IL-6, and TNF-*α*. Growing evidences and clinical trials indicate that anticytokine biologic agents, e.g., anakinra (interleukin-1 receptor antagonist) [46] and tocilizumab (anti-IL-6 receptor antibody) [47], are becoming plausible therapeutic options for the management of AOSD. Recently, Jaguin et al. reported the phenotypic and genomic markers of M-CSF-generated human macrophages polarized towards M1 or M2 subtype upon the action of lipopolysaccharide and interferon-*γ* (for M1) or interleukin- (IL-) 4 (for M2) [48]. The ability of human M-CSF-generated macrophages to polarize toward M1 or M2 subtype was associated with enhanced secretion of TNF-*α*, IL-1*β*, IL-12p40, CXCL10, and IL-10 (for M1) or CCL22 (for M2) [48]. In addition, Bellora et al. reported that M-CSF could induce the expression of membrane-bound IL-18 in human blood monocytes differentiating toward macophages [49]. The enhancement of these macrophage-triggered cytokines was frequently observed in AOSD patients [22, 50]. This functional property of M-CSF highlights the innate immunity and subsequent adaptive immune responses implicated in AOSD pathogenesis. In this study, we report that the novel SNP rs11102024 near *CSF1* gene encoding for M-CSF is highly associated with AOSD and links to higher plasma levels of M-CSF, which would be a potential and promising therapeutic target for AOSD.

## 5. Conclusion

Adult-onset Still's disease (AOSD) is a rare, but systemic inflammatory disorder characterized by spiking fever, rash, leukocytosis, arthritis, and multisystemic involvement, with increased macrophages and M-CSF. Herein, we identify a novel SNP rs11102024 on the 5′-upstream of *CSF1* gene to be significantly associated with AOSD in a GWAS and subsequent replication analysis. Reflecting the function of *CSF1*, encoding M-CSF which is involved in macrophage differentiation and inflammatory responses, the SNP rs11102024 demonstrated the significant association with plasma M-CSF levels, disease activity, and disease outcome of AOSD. These data suggest that the gene variant rs11102024 nearby *CSF1* could be a potential prognostic factor for a disease outcome in AOSD. These results provide a promising initiative to predict and early intervention to improve the treatment and healthcare for AOSD. Further investigation is needed to a better understanding of the pathogenesis of AOSD and clinical implementation for the genetic variants of *CSF1*.

## Figures and Tables

**Table 1:** Demographic data, clinical characteristics, and laboratory findings of 106 patients with adult-onset Still's disease (AOSD).

Characteristics^∗^	AOSD patients
Age at study entry (mean ± SD, years)	42.9 ± 15.0
Females, *n* (%)	72 (67.9%)
Fever (≧39°C), *n* (%)	102 (96.2%)
Evanescent rash, *n* (%)	94 (88.7%)
Arthralgia or arthritis, *n* (%)	78 (73.6%)
Sore throat, *n* (%)	74 (69.8%)
Liver dysfunction, *n* (%)	42 (39.6%)
Lymphadenopathy, *n* (%)	39 (36.8%)
Hepatosplenomegaly, *n* (%)	14 (13.2%)
White blood cell count (mean ± SD, cells/microliter)	16205 ± 11673
Platelet count (mean ± SD, ×10^3^/cumm)	351.5 ± 151.0
ESR values (mean ± SD, mm/1^st^ hour)	78.4 ± 36.6
CRP levels (mean ± SD, mg/dL)	10.2 ± 7.8
Ferritin levels (mean ± SD, *μ*g/L)	8989 ± 15328
Clinical activity score (mean ± SD)	5.48 ± 1.11

^∗^Data are presented as the mean ± standard deviation (SD) or number (percentage). Liver dysfunction was defined as alanine aminotransferase (ALT) level≧40 IU/L. ESR: erythrocyte sedimentation rate; CRP: C-reactive protein.

**Table 2:** Association between the top 4 significant SNPs and AOSD in the initial GWAS screen.

rs ID	Nearby gene	Chromosome position	Location	Minor allele	AOSD cases (*n* = 70) Genotype AA/AB/BB	Controls (*n* = 688) Genotype AA/AB/BB	*P* value^1^	Odds ratio^1^ (95% CI)	HWE *P* value^2^	Call rate^3^
					MAF	MAF				
rs11102024	*CSF1*	1 : 110431514	5′UTR	T	47/14/9	587/97/3	3.70 × 10^−7^	3.27 (2.07~5.17)	0.64	99.9%
					22.9	7.5				
rs35910146	*RPL3P8* (pseudogene)	7 : 109659225	Upstream	T	28/33/8	468/186/15	1.33 × 10^−7^	3.11 (2.04~4.75)	0.49	97.4%
					35.5	16.1				
rs6948305	*LOC100419782* (pseudogene)	7 : 109757108	Upstream	G	30/32/8	477/197/14	6.43 × 10^−7^	2.93 (1.92~4.47)	0.22	100%
					34.3	16.4				
rs9636107	*TCF4*	18 : 53200117	Intron	G	35/30/5	525/159/4	6.87 × 10^−7^	3.35 (2.08~5.40)	0.03	100%
					28.6	12.1				

The genomic coordinates are based on NCBI Human Genome Build 37 (GRCh37). Gene ID: *CSF1* (colony-stimulating factor 1; NCBI Entrez Gene 1435), *RPL3P8* (ribosomal protein L3 pseudogene 8; NCBI Entrez Gene 646620), *LOC100419782* (zinc finger protein 717 pseudogene; NCBI Entrez Gene 100419782), and *TCF4* (transcription factor 4; NCBI Entrez Gene 6925). MAF: minor allele frequency; 95% CI: 95% confidence interval; 5′UTR: 5′ untranslated region. ^1^*P* values and odds ratio were derived with a logistic regression model adjusted by sex and principal components (PCs) in 70 AOSD cases and 688 population controls. ^2^Hardy-Weinberg equilibrium (HWE), *P* values for 688 controls from the general population. ^3^Cell rate is for overall samples (70 AOSD and 688 controls).

**Table 3:** Association of rs11102024 with adult-onset Still's disease (AOSD) in the GWAS discovery, replication, and combined sample analyses.

		GWAS discovery (70 AOSD cases versus 688 population controls)				Replication analysis (36 AOSD cases versus 200 population controls)				Combined sample analysis (106 AOSD cases versus 888 population controls)			
SNP	Allele	MAF of cases	MAF of controls	*P* value^1^	Odds ratio^1^ (95% CI)	MAF of cases	MAF of controls	*P* value^2^	Odds ratio (95% CI)	*P* value^2^	Odds ratio (95% CI)	HWE^3^	*P*_het_^4^
rs11102024	A/T	22.9	7.5	3.70 × 10^−7^	3.27 (2.07~5.17)	16.7	7.5	0.022	2.47 (1.20~5.08)	1.20 × 10^−8^	3.28 (2.25~4.79)	0.33	0.36

MAF: minor allele frequency; OR: odds ratio; 95% CI: 95% confidence interval. ^1^*P* value and odds ratio were derived by logistic regression adjusted with sex and principal components (PCs) from the GWAS discovery result. ^2^*P* values were calculated by Fisher's exact test for the risk allele. ^3^Hardy-Weinberg equilibrium (HWE); *P* values for 888 controls from the general population. ^4^^.^*P*_het_: *P* value of the heterogeneity test between studies.

## Data Availability

All data relevant to the study are included in the article or uploaded as supplementary information.

## Abbreviations

AOSD: Adult-onset Still's disease
CI: Confidence interval
*CSF1*: Colony-stimulating factor 1
GWAS: Genome-wide association study
ELISA: Enzyme-linked immunosorbent assay
HWE: Hardy-Weinberg equilibrium
LD: Linkage disequilibrium
MAF: Minor allele frequency
M-CSF: Macrophage colony-stimulating factor
OR: Odds ratio
PCA: Principal component analysis
SNP: Single-nucleotide polymorphism
UTR: Untranslated region.

## Supplementary Materials

Supplementary Materials Supplemental Table 1: association between SNPs on HLA region and AOSD. Supplemental Table 2: proxy variants in linkage disequilibrium (LD) with the SNP 11102024. Supplemental Figure 1: a scatter plot of the principal components analysis (PCA) for the two principal components (PC1 and PC2). Supplemental Figure 2: a quantile-quantile plot of the test statistics in a case-control study for AOSD. Supplemental Figure 3: linkage disequilibrium (LD) analysis of SNP rs11102024 in the East Asian (EAS) population.
