# Supplementary material for: Genetic Association and Expression Correlation between Colony-Stimulating Factor 1 Gene Encoding M-CSF and Adult-Onset Still's Disease
Source: J Immunol Res. 2020 Feb 14;2020:8640719. doi: 10.1155/2020/8640719 (PMC7042538; doi:10.1155/2020/8640719)
Supplement: Supplementary Materials — Supplemental Table 1: association between SNPs on HLA region and AOSD. Supplemental Table 2: proxy variants in linkage disequilibrium (LD) with the SNP 11102024. Supplemental Figure 1: a scatter plot of the principal components analysis (PCA) for the two principal components (PC1 and PC2). Supplemental Figure 2: a quantile-quantile plot of the test statistics in a case-control study for AOSD. Supplemental Figure 3: linkage disequilibrium (LD) analysis of SNP rs11102024 in the East Asian (EAS) population. [file 8640719.f1.doc]

**Supplemental Material**

**Genetic association and expression correlation between colony-stimulating factor 1 gene encoding M-CSF and Adult-onset Still’s disease**

Yi-Ming Chen^1,2,3,§^, Wei-Ting Hung^1,4,§^, Wan-Chun Chang^5^, Chia-Wei Hsieh^1,3^, Wen-Hung Chung^5,6,7,8^, Joung-Liang Lan^9,10,11^, Ning-Rong Gung^9^, , Yun-Shien Lee^12^, Der-Yuan Chen^9,10,13*^, Shuen-Iu Hung^5,6,7,8,14*^

Supplemental Table 1: Association between SNPs on *HLA* region and AOSD

Supplemental Table 2: Proxy variants in linkage disequilibrium (LD) with the SNP 11102024

Supplemental Figure 1: A Scatter plot of the principal components analysis (PCA) for the two principal components (PC1 and PC2).

Supplemental Figure 2: A quantile-quantile plot of the test statistics in case-control study for AOSD.

Supplemental Figure 3: Linkage disequilibrium (LD) analysis of SNP rs11102024 in the East Asian (EAS) population.

**Supplemental Table 1. Association between SNPs on *HLA* region and AOSD**

| **Chromosome location** | **SNP** | **Nearby gene (Gene ID)** | ***P* value** |
| --- | --- | --- | --- |
| 6: 31099577 | rs4959053 | PSORS1C1 (170679) | 0.0006095 |
| 6: 32360488 | rs7744293 | HCG23 (414764) | 0.0006219 |
| 6: 32389255 | rs3135365 | **HLA-DRA** (3122) | 0.0003555 |
| 6: 32574060 | rs9270986 | **HLA-DRB1** (3123) | 0.0004241 |
| 6: 32788511 | rs9784758 | --- | 8.24E-05 |
| 6: 34083564 | rs13196449 | GRM4 (2914) | 0.0008644 |

The genomic coordinates are based on NCBI Human Genome Build 37 (GRCh37). *P* values were derived with the logistic regression model adjusted by sex and principal components (PCs) in 70 AOSD cases and 688 population controls.

**Supplemental Table 2. Proxy variants in linkage disequilibrium (LD) with the SNP 11102024**

| **RS Number** | **Chr** | **Position (GRCh37)** | **Alleles** | **MAF** | **Distance** | **D'** | **R^2^** | **Correlated Alleles** | **RegulomeDB**^†^ | **Functional Class** |
| --- | --- | --- | --- | --- | --- | --- | --- | --- | --- | --- |
| [rs34119694](http://www.ncbi.nlm.nih.gov/projects/SNP/snp_ref.cgi?rs=34119694) | 1 | [110430650](http://genome.ucsc.edu/cgi-bin/hgTracks?db=hg19&position=chr1%3A110430400-110430900&snp151=pack&hgFind.matches=rs34119694) | (G/A) | 0.0952 | 864 | 1 | 0.9885 | A=G,T=A | [4](http://www.regulomedb.org/snp/chr1/110430649) | NA |
| [rs77573287](http://www.ncbi.nlm.nih.gov/projects/SNP/snp_ref.cgi?rs=77573287) | 1 | [110430656](http://genome.ucsc.edu/cgi-bin/hgTracks?db=hg19&position=chr1%3A110430406-110430906&snp151=pack&hgFind.matches=rs77573287) | (C/T) | 0.0952 | 858 | 1 | 0.9885 | A=C,T=T | [4](http://www.regulomedb.org/snp/chr1/110430655) | NA |
| [rs418976](http://www.ncbi.nlm.nih.gov/projects/SNP/snp_ref.cgi?rs=418976) | 1 | [110430929](http://genome.ucsc.edu/cgi-bin/hgTracks?db=hg19&position=chr1%3A110430679-110431179&snp151=pack&hgFind.matches=rs418976) | (G/C) | 0.0952 | 585 | 1 | 0.9885 | A=C,T=G | [7](http://www.regulomedb.org/snp/chr1/110430928) | NA |
| [rs74649113](http://www.ncbi.nlm.nih.gov/projects/SNP/snp_ref.cgi?rs=74649113) | 1 | [110431147](http://genome.ucsc.edu/cgi-bin/hgTracks?db=hg19&position=chr1%3A110430897-110431397&snp151=pack&hgFind.matches=rs74649113) | (G/A) | 0.0952 | 367 | 1 | 0.9885 | A=G,T=A | [6](http://www.regulomedb.org/snp/chr1/110431146) | NA |
| [rs376296](http://www.ncbi.nlm.nih.gov/projects/SNP/snp_ref.cgi?rs=376296) | 1 | [110431312](http://genome.ucsc.edu/cgi-bin/hgTracks?db=hg19&position=chr1%3A110431062-110431562&snp151=pack&hgFind.matches=rs376296) | (T/C) | 0.0942 | 202 | 1 | 1 | A=C,T=T | [6](http://www.regulomedb.org/snp/chr1/110431311) | NA |
| [rs11102023](http://www.ncbi.nlm.nih.gov/projects/SNP/snp_ref.cgi?rs=11102023) | 1 | [110431391](http://genome.ucsc.edu/cgi-bin/hgTracks?db=hg19&position=chr1%3A110431141-110431641&snp151=pack&hgFind.matches=rs11102023) | (A/C) | 0.0942 | 123 | 1 | 1 | A=C,T=A | [6](http://www.regulomedb.org/snp/chr1/110431390) | NA |
| **rs11102024** | 1 | 110431514 | (A/T) | 0.085 | 0 | --- | --- | --- | --- | NA |
| [rs427760](http://www.ncbi.nlm.nih.gov/projects/SNP/snp_ref.cgi?rs=427760) | 1 | [110432467](http://genome.ucsc.edu/cgi-bin/hgTracks?db=hg19&position=chr1%3A110432217-110432717&snp151=pack&hgFind.matches=rs427760) | (G/A) | 0.0952 | 953 | 1 | 0.9885 | A=A,T=G | [6](http://www.regulomedb.org/snp/chr1/110432466) | NA |
| [rs76230932](http://www.ncbi.nlm.nih.gov/projects/SNP/snp_ref.cgi?rs=76230932) | 1 | [110433453](http://genome.ucsc.edu/cgi-bin/hgTracks?db=hg19&position=chr1%3A110433203-110433703&snp151=pack&hgFind.matches=rs76230932) | (C/T) | 0.0962 | 1939 | 0.9884 | 0.9546 | A=C,T=T | [4](http://www.regulomedb.org/snp/chr1/110433452) | NA |
| [rs333953](http://www.ncbi.nlm.nih.gov/projects/SNP/snp_ref.cgi?rs=333953) | 1 | [110433623](http://genome.ucsc.edu/cgi-bin/hgTracks?db=hg19&position=chr1%3A110433373-110433873&snp151=pack&hgFind.matches=rs333953) | (A/G) | 0.0972 | 2109 | 1 | 0.9662 | A=G,T=A | [5](http://www.regulomedb.org/snp/chr1/110433622) | NA |
| [rs75354050](http://www.ncbi.nlm.nih.gov/projects/SNP/snp_ref.cgi?rs=75354050) | 1 | [110434301](http://genome.ucsc.edu/cgi-bin/hgTracks?db=hg19&position=chr1%3A110434051-110434551&snp151=pack&hgFind.matches=rs75354050) | (T/G) | 0.1002 | 2787 | 1 | 0.9344 | A=T,T=G | [7](http://www.regulomedb.org/snp/chr1/110434300) | NA |
| [rs79460648](http://www.ncbi.nlm.nih.gov/projects/SNP/snp_ref.cgi?rs=79460648) | 1 | [110435039](http://genome.ucsc.edu/cgi-bin/hgTracks?db=hg19&position=chr1%3A110434789-110435289&snp151=pack&hgFind.matches=rs79460648) | (C/T) | 0.0923 | 3525 | 0.8575 | 0.7183 | A=C,T=T | [4](http://www.regulomedb.org/snp/chr1/110435038) | NA |

*Linkage disequilibrium (LD) analysis of the SNP rs11102024 was calculated by using the LDproxy module of the online software package LDLink ([**https://analysistools.nci.nih.gov/LDlink**](https://analysistools.nci.nih.gov/LDlink)).^1^

†RegulomeDB score as defined at **[http://www.regulomedb.org/help#score](http://www.regulomedb.org/help#score)**. The scoring scheme refers: 4, TF binding + DNase peak; 5, TF binding or DNase peak; 6, other; 7, no annotation.

Reference:

1 Machiela MJ, Chanock SJ. LDlink: a web-based application for exploring population-specific haplotype structure and linking correlated alleles of possible functional variants. *Bioinformatics* 2015;31:3555–7.


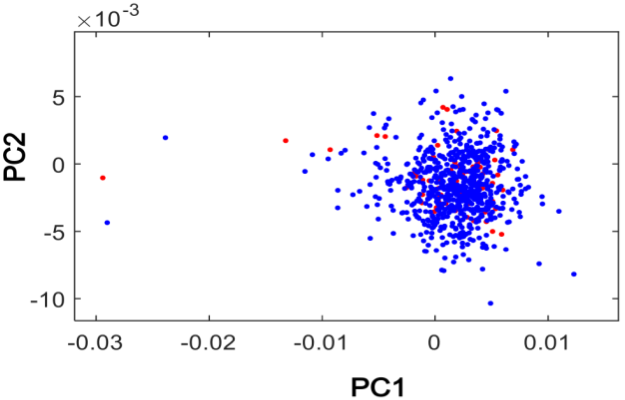


Supplemental Figure 1. A Scatter plot of the principal components analysis (PCA) for the two principal components (PC1 and PC2). The PCA was performed using the data obtained from Affymetrix SNPs Array 6.0 platform including 645,983 SNPs on chromosome 1 to chromosome 22 of 70 AOSD patients (labeled by red dots) and 688 general population-controls (labeled by blue dots) from Taiwan. The PCA plot cannot separate the 70 AOSD cases from 688 general controls, suggesting that there is no population stratification between the cases and controls.


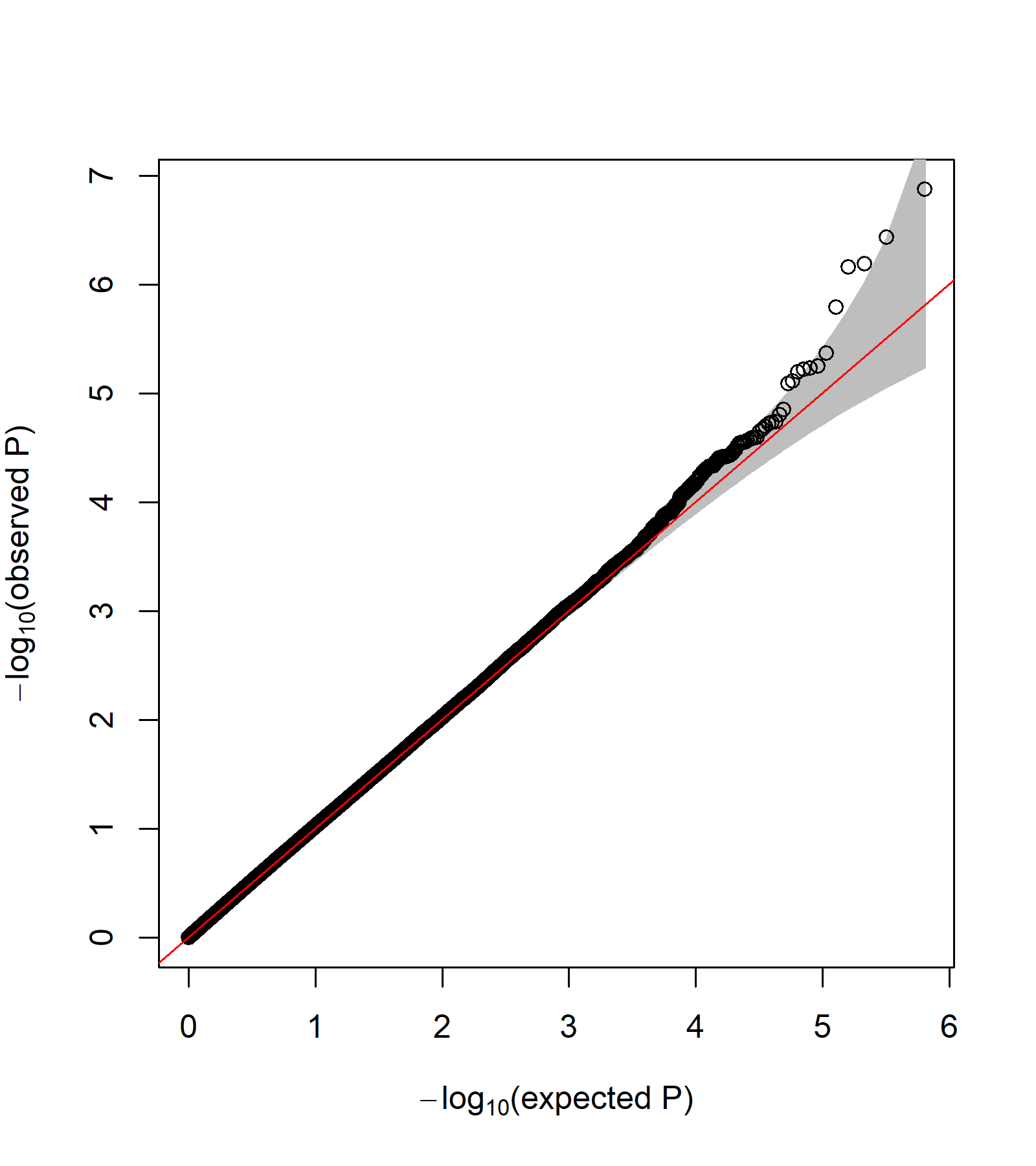


Supplemental Figure 2. A quantile-quantile plot of the test statistics in case-control study for AOSD. The theoretical quantile-quantile plot of the χ2 statistics in the case-control study is shown. The red line represents the null model, in which the observed χ2 values match the expected values. The gray chunk represents the 95% confidence interval of the null model. The black dots represent the observed χ2 values compared with the expected values from the case-control study. When the SNPs located in the gray region indicates that the p-value deviates from the expected distribution.

**
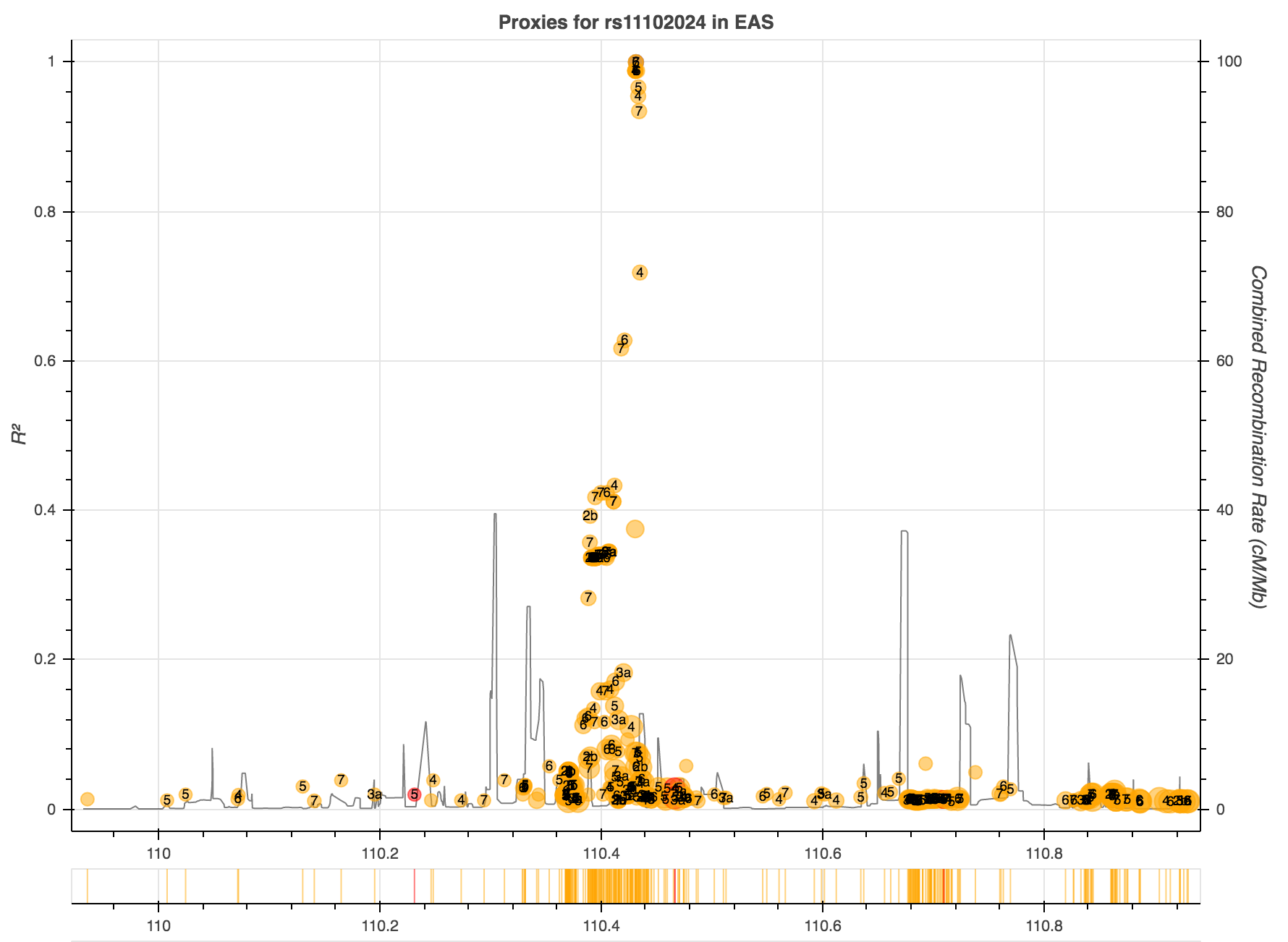
**

**
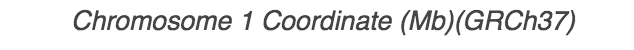
**

**
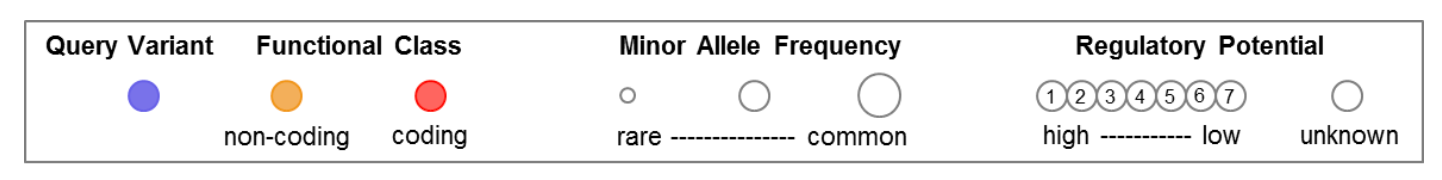
**

**Supplemental Figure 3.** Linkage disequilibrium (LD) analysis of SNP rs11102024 in the East Asian (EAS) population. Linkage of the SNP rs11102024 was calculated by using the LDproxy feature of the online software package LDLink (<https://analysistools.nci.nih.gov/LDlink>).^1^ The proxy algorithm data from LDproxy module was simulated using realistic linkage disequilibrium patterns obtained from the 1,000 Genomes project.

Reference:

1 Machiela MJ, Chanock SJ. LDlink: a web-based application for exploring population-specific haplotype structure and linking correlated alleles of possible functional variants. *Bioinformatics* 2015;31:3555–7.
